# Video Head Impulse test findings in young adults with long-term use of Personal Listening Devices

**DOI:** 10.1007/s00405-025-09831-w

**Published:** 2026-01-06

**Authors:** Teja Deepak Dessai, Jiss Mariya Sunny, Rashmi J. Bhat, Kaushlendra Kumar

**Affiliations:** 1 Manipal Academy of Higher Education, Manipal, India; 2 Bangalore Speech and Hearing Research Foundation, Bangalore, India; 3 Dr. S. R. Chandrasekhar Institute of Speech and Hearing, Bangalore, India; 4 Department of Audiology and Speech Language Pathology, Kasturba Medical College Mangalore, Manipal Academy of Higher Education, Manipal, India

**Keywords:** vHIT, Personal Listening Devices, Vestibular System, VOR

## Abstract

**Purpose:**

The prolonged use of personal listening devices (PLDs) at high volumes may impact both hearing and balance. This study aimed to investigate whether high-volume PLD use leads to early vestibular changes detectable through the video head impulse test (vHIT) in normal-hearing individuals.

**Methods:**

A total of 100 participants aged 15–24 years with normal hearing and PLD use at >60% of the maximum volume were included. They were divided into two groups: control (>1 year of PLD use) and experimental (>1 year of PLD use). Output sound levels near the tympanic membrane were measured using the microphone-in-real-ear (MIRE) method, followed by vHIT assessment of all six semicircular canals. The parameters analysed included vestibulo-ocular reflex (VOR) gain, precision rate (PR) score, and covert saccades. The data were analysed via descriptive statistics and the Mann–Whitney U test.

**Results:**

The results revealed that the experimental group listened to significantly higher PLD output levels, but no significant differences were observed in the vHIT parameters between the groups.

**Conclusions:**

All findings remained within normal clinical limits. The results suggest that prolonged PLD use at moderately high volumes does not cause measurable semicircular canal dysfunction. However, continued promotion of safe listening practices remains essential for long-term auditory and vestibular health.

## Introduction

The use of personal listening devices (PLDs) among younger generations has been associated with hearing difficulties. A study by Peng et al. [1] revealed altered hearing thresholds in the 3–8 kHz frequency range, which further expanded with prolonged exposure to PLDs at higher intensity levels. Similarly, a study conducted by Le Prell et al. [2] reported that listening to PLDs at higher volume levels can cause subtle preclinical damage to the auditory system and vestibular end organs, leading to potential hazardous effects on hearing and balance systems.

Exposure to loud sounds can cause anatomical and physiological changes in the auditory and vestibular systems. Stewart [3] confirmed that cellular damage throughout the peripheral vestibular system was caused by noise exposure. There is a close connection between the cochlea and vestibular sensory end organs, both of which are housed within the temporal bone and membranous labyrinth of the inner ear. Santos et al. [4] reported an association between hearing loss and vestibular disorders, highlighting that changes in one system can cause significant damage to the other. Additionally, Yamamoto et al. [5] reported that constant noise overstimulation can damage the saccular membrane before affecting the Reissner's membrane, increasing the susceptibility of the former to noise-induced damage. Yilmaz [6] also reported a significant loss of Vestibulo-ocular Reflex (VOR) gain in individuals with noise-induced hearing loss, indicating potential vestibular dysfunction caused by prolonged exposure to PLDs at high volume levels.

The exposure to loud intensities can result in various anatomical and physiological changes, including missing hair cells, supporting cells, and nerve fibres, buckling of the pillar bodies, temporary threshold shifts (TTS) averaging 43 dB in the 4–12 kHz range, and alterations in the embedding of outer hair cell (OHC) stereocilia in the tectorial membrane during TTS. Moreover, permanent threshold shifts (PTSs) with focal losses of inner and outer hair cells and afferent nerve fibres at specific frequency locations are observed [7].

Hair cells, which are the sensory cells of the inner ear, have similar morphologies in the vestibular end organs and the organ of Corti in the cochlea. Both systems transduce hair bundle displacement into neural activity through the shared vestibulocochlear nerve (CN VIII) [3]. Furthermore, constant noise overstimulation can damage the saccular membrane before the Reissner's membrane, as the former has lower tensile strength and breakage points, predisposing it to noise-induced damage [5]. This suggests that exposure to PLDs may lead to vestibular dysfunction even before cochlear dysfunction occurs, highlighting the importance of including a vestibular test battery in clinical setups.

The video head impulse test (vHIT) is an objective test used to assess VOR. The VOR stabilizes eye gaze while the head is moving by activating the vestibular system and coordinating eye movements [8]. The vHIT evaluates the VOR function of all three semicircular canals involved in maintaining balance and plays a crucial role in gaze stabilization by producing compensatory eye movements that maintain a stable visual image during rapid head movements. It examines the VOR in pairs, including the left-anterior-right-posterior (LARP) canals, right-anterior/left-posterior (RALP) canals, and left lateral-right lateral (LLRL) canals. The incorporation of vHIT testing can (a) rule out vestibular dysfunction in individuals using PLDs, (b) improve the recordability of responses and enhance the overall sensitivity of diagnostic test batteries for identifying vestibular impairments, (c) serve as an educational material to increase awareness, (d) be utilized for counselling and rehabilitating individuals experiencing vestibular symptoms due to PLD usage, and (e) contribute to enhancing the quality of life of affected individuals. Thus, the aim of this study was to describe the vHIT findings in individuals with normal hearing and prolonged exposure to PLDs at more than 60% volume.

## Method

This cross-sectional study was carried out after obtaining ethical clearance from the Institutional Ethical Committee for carrying out the study.

Initially, 334 individuals were screened via a questionnaire designed to gather information about their PLD usage over the years. Of these, 217 individuals met the study's inclusion criteria. Among them, 121 consented to participate, and finally, 100 individuals were recruited through convenience sampling. All the participants within the age range of 15–24 years were recruited for this study via a convenient sampling method and were divided into two groups: a control group and an experimental group. The control group comprised 50 participants who used PLDs for less than one year at a volume level greater than 60% of the device's maximum. The experimental group consisted of 50 participants who had used PLDs for more than one year at a volume level exceeding 60% of the device's maximum. All participants provided written informed consent without any cost to participate in the study, and their selection was rigorously based on predefined inclusion and exclusion criteria.

All assessments were conducted in a room adhering to American National Standards Institute [9] standards for lighting and soundproofing, ensuring optimal testing conditions. Each recruited participant underwent a comprehensive battery of tests for both ears, starting with otoscopy via a Welch Allyn otoscope to visually examine the external ear canal and tympanic membrane. Next, puretone audiometry was performed via a calibrated dual-channel Grason Stadler Inc. Audio star instrument with TDH-39 headphones and a radioear B-71 bone vibrator, which involved both air conduction testing for frequencies ranging from 250 Hz-8000 Hz and bone conduction testing for frequencies ranging from 250 Hz-4000 Hz. Participants with thresholds < 15 dBHL in both air conduction and bone conduction were considered eligible. Both tympanometry and Reflexometry were also performed via a calibrated Grason Stadler Inc. GSI Tympstar Pro middle ear analyser. Tympanometry utilized a 226 Hz probe tone, and acoustic reflexes were elicited in both ears for each participant at 500 Hz, 1000 Hz, 2000 Hz, and 4000 Hz. Only individuals exhibiting an 'A' type tympanogram and presenting acoustic reflexes (both ipsilateral and contralateral) were ultimately recruited for the study. The exclusion criteria included participants with a history of any middle ear or vestibular pathology; those trained in athletics, gymnastics, dancers, or yoga practitioners; and those who had taken immune suppressants and/or vestibule-toxic agents within 48 h before testing. Additionally, participants who experienced neck or shoulder pain were excluded.

The dBSPL levels used by participants with PLDs were subsequently calculated near the tympanic membrane. A microphone in real ear (MIRE) adopted from Singh and Sasidharan [10] was used. The obtained dBSPLs were then converted to dBA values for comparison with the occupational noise exposure damage risk criterion (DRC). This comparison aimed to determine whether PLD exposure could lead to negative effects similar to those caused by occupational noise exposure. The participants subsequently underwent vHIT.

The participants were asked to sit in a static chair in a well-lit room. A target at eye level was fixed at a minimum of one meter in front of it. The participants were told to keep their eyes wide open, relax their necks, and concentrate on the target dot in front of them. The vestibular function of each of the three semicircular canals was measured via video goggles (ICS Impulse, Otometrics, Denmark). This goggle features a laser light, a motion sensor that detects head movement, and an integrated video camera that captures eye movement in real time. The vHIT goggles were adjusted to the wearer's head until the least amount of movement was detected at the nasal bridge. To prevent goggles from slipping off participants' heads, an elastic band that could be tightened or loosened was used to secure each participant's goggles. To guarantee that head motions would be precisely recorded by the eye, the camera location was changed to focus the pupil on the video display. Calibration was performed via a laser affixed to the center of the goggles. The calibration procedure was completed under the same room lighting conditions as the vHIT. It included two distinct eye calibration procedures for horizontal and vertical canal testing. Each participant completed the calibration process before proceeding with the actual testing. Next, the participants were instructed to fixate on the target on the wall, whereas the primary investigator stood behind the participants and performed 20 unpredictable, low-amplitude (10–20°) head impulses for each side and for all three canal planes (LLRL, LARP and RALP); in general, the horizontal head velocities were higher (150–250°/s) than the vertical head velocities (50–150°/s). For the horizontal plane, the primary investigator placed their hands on the participant’s jaw, whereas for the vertical plane, the dominant hands were placed on the top of the head and the other under the chin. For the right/left horizontal canal plane, horizontal head turns were made to each side, always beginning in the middle. For the RALP canal test, the subject’s head was rotated 45° to the left, and the impulses were administered by moving the head either forward or backwards while constantly asking the patient to focus on the object. On the other hand, in the LARP canal test, the individual turns 45° to the right, and impulses are administered by moving the head either forward or backwards [11]. The study noted two key parameters for each participant: VOR gain and precision rate (PR) score. The PR score is the numerical score that quantifies the number of scattered saccadic responses. A higher PR score indicates scattered grouped responses, and a lower PR score indicates maximum gathered responses [12]. In the case of a normal ear with no balance-related symptoms, the PR score is lower [13]. Similarly, the percentage of covert saccades were also checked. These parameters were assessed in all three canal testings.

## Results

The primary objective of this study was to outline the findings of the vHIT in individuals with normal hearing and prolonged exposure to PLDs at volumes exceeding 60%. A total of 100 participants were enrolled in the study and categorized into two groups on the basis of the inclusion and exclusion criteria. Both the experimental group and the control group comprised 50 participants each, with ages ranging from 15–24 years.

In light of the study's objectives, the examination initially focused on comparing the output sound levels (in decibels, dBA) near the tympanic membrane from PLDs between the experimental and control groups. Various vHIT parameters, including VOR gain, PR score, and the presence of covert saccades, were subsequently compared between these two groups. The findings of the study are mentioned below.

For data analysis, SPSS version 20 software (2011) was utilized. Initial assessments involved applying the Kolmogorov‒Smirnov and Shapiro‒Wilk tests to evaluate the normality of the collected data. A test of normality was conducted for the output dBA level measurement data, and the results indicated a normal distribution. Consequently, a parametric test was used to assess the significant difference between the experimental and control groups for these data. Conversely, a test of normality for the vHIT data revealed that all the data variables were not normally distributed (*p* < 0.05), with the sole exception of lateral canal VOR gain (*P* > 0.05). Therefore, a nonparametric test was conducted to assess the significant difference in means between the experimental and control groups for the vHIT data.

### Output dBA level from PLDs near the tympanic membrane

The measurement of the dBA sound levels produced by PLDs in close proximity to the tympanic membrane was carried out via MIRE. A probe microphone was carefully inserted into the ear canal to capture the output dBA levels. The participants were then guided to adjust the PLD’s volume settings to match their typical listening levels. Descriptive statistics were subsequently applied to analyse the mean output dBA values obtained from the PLDs positioned near the tympanic membrane in both the control and experimental groups. Table 1 indicates that the experimental group listened to a higher volume level than did the control group.

**Table 1:** Mean output dBA values from PLDs in both the control and experimental groups

Group	No: of participants (*n*)	Minimum (dBA)	Maximum (dBA)	Mean (dBA)	SD
Control	50	40.68	74.01	56.77	7.95
Experimental	50	55.59	90.01	69.60	7.88

Additionally, an independent t test was conducted to compare the mean output sound levels of the PLDs, measured near the tympanic membrane via MIRE, between the experimental and control groups. This analysis revealed a statistically significant difference in these mean output levels across the two groups (t (98) = -8.105, *p* < 0.0001).

**Fig. 1:**
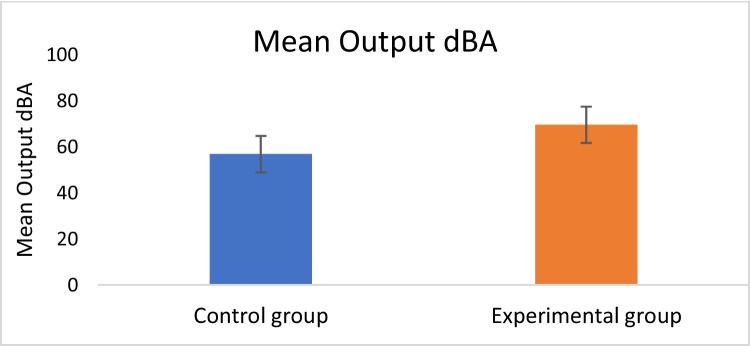
Mean dBA output from the PLDs near the tympanic membrane between the control and experimental groups

Figure 1 shows the mean output dBA levels from the PLDS near the tympanic membrane between the experimental and control groups.

Subsequently, statistical analyses via the Wilcoxon signed-rank test were conducted on the obtained VOR gain values. A comparison of VOR gain between the right and left ears revealed no significant difference (*p* > 0.05) across the two groups. Hence, further analyses were carried out by considering the total number of ears in the control and experimental groups. The Mann‒Whitney U test was conducted to assess the significant difference in the mean scores of VOR gain between the control and experimental groups. The results indicate that there is no significant difference in the mean scores between the groups since the p value was greater than the preferred significance level of 5%. The mean VOR gain obtained from the descriptive statistics for both groups is presented in Table 2.

**Table 2:** Mean VOR gain in the control and experimental groups

Group	Number of participants	Number of ears	Lateral Semicircular canals	Anterior Semicircular canals	Posterior Semicircular canals
			Mean	Mean	Mean
Control	50	100	0.92 ± 0.09	0.92 ± 0.13	0.92 ± 0.17
Experimental	50	100	0.92 ± 0.08	0.92 ± 0.15	0.91 ± 0.11

**Fig. 2:**
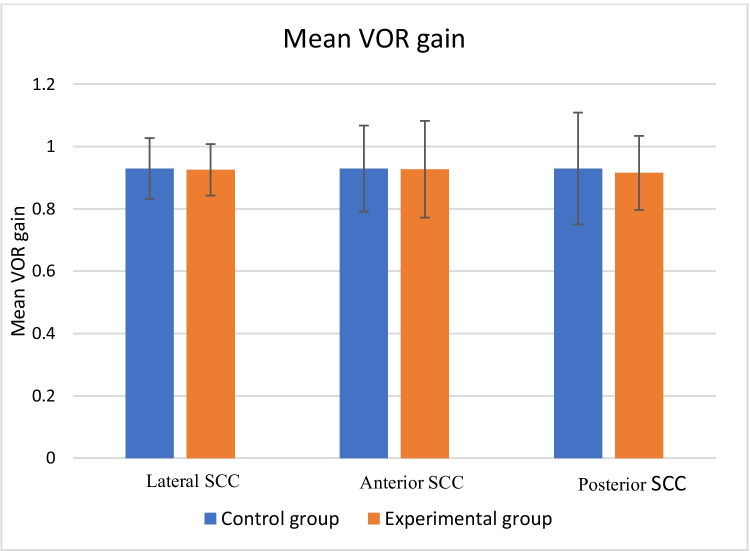
Mean VOR gain between the control and experimental groups

**Fig. 3:**
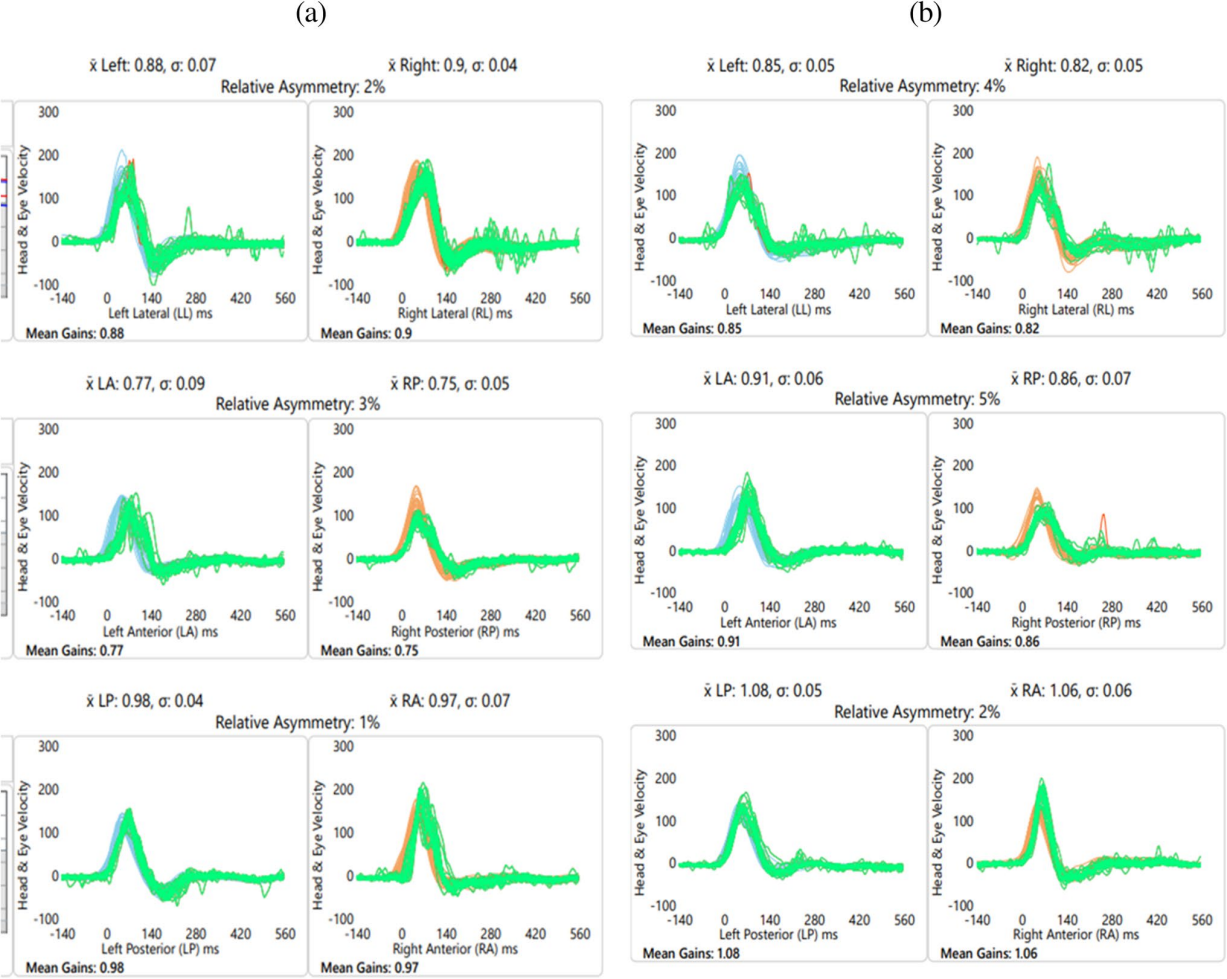
Graphical representation of the VOR gain of the (**a**) control group and (**b**) experimental group

Figure 2 depicts the mean VOR gain between the control and experimental groups. Similarly, Fig. 3 illustrates the graphical representation of VOR gain for (a) the control group and (b) the experimental group.

The mean PR score obtained from the descriptive statistics of both the experimental and control groups are depicted in Table 3.

Table 3 shows nearly the same mean PR scores for both the experimental and control groups. The Mann‒Whitney U test was conducted to assess the significant differences in the mean PR score between the control and experimental groups. The results indicate that there was no significant difference in the mean scores between the groups (*p* > 0.05). Similarly, corrective saccades were observed in less than 10% of impulses for both ears across all three canal tests.

**Table 3:** Mean PR score in the control and experimental groups

Group	Number of participants	Number of ears	PR score		
			Lateral SCC	Anterior SCC	Posterior SCC
Control	50	100	3.52 ± 12.02	2.38 ± 9.05	2.02 ± 7.66
Experimental	50	100	3.50 ± 8.80	2.37 ± 7.53	2.04 ± 5.65

## Discussion

Notably, a 100% response rate was achieved from both groups for the tests conducted among the included participants. The participants in the experimental group, who had used PLDs for more than one year, listened to significantly higher output sound levels (mean: 69.60 ± 7.88 dBA) compared to the control group (< 1 year of use, mean: 56.77 ± 7.95 dBA). In a similar vein, Kim et al. [14] have reported PLD listening level between 57.32 dBA to 83.43 dBA among college students. These listening levels do not exceed the DRC used for occupational hearing safety levels set by regulatory bodies.

VOR gain was measured following the procedure described by Halmagyi and Curthoys [15], with 20 valid trials performed for each semicircular canal. Across both groups, VOR gain values were consistent with normative ranges reported in healthy individuals (lateral canals: 0.8–1.2; vertical canals: 0.7–1.2) [16–22]. No statistically significant difference in the mean VOR gain was found between the experimental and control groups, suggesting that prolonged PLD use at the measured dBA levels does not impact semicircular canal function.

The absence of significant differences in vHIT parameters may be attributed to the relatively low sound pressure levels (SPLs) used by participants. Among the 50 participants only two participants in the experimental group exceeded the DRC of 85 dBA for 8 h per day, as stipulated by national and international standards, including references such as the NIOSH [23], the Indian Ministry of Environments and Forest [24], and the International Standard [25]. The remaining participants were exposed to PLD levels below the DRC, suggesting that their auditory and vestibular damage may not be at risk due to this exposure.

Previous research has shown that high-intensity noise exposure can harm cochlear and vestibular structures. Experimental and clinical studies suggest that loud noise can stimulate vestibular end-organs, causing dysfunction.For example, Ylikoski et al. [26] reported a higher incidence of vestibular dysfunction in soldiers. Collins [27] similarly reported labyrinthine dysfunction in 54% and labyrinthine lesions in 41% of the 108 individuals studied. Numerous reports have also demonstrated that industrial workers with noise-induced hearing loss frequently experience balance disorders [28]. Furthermore, Shupak et al. [29] reported significantly lower VOR gains in military personnel and industrial workers with noise-induced hearing loss than in a normal-hearing control group.

Research on noise-induced vestibular damage, particularly from PLDs, reveals varying effects on the basis of exposure level and duration [1]. Singh and Sasidharan [5] reported reduced P1N1 peaks in cervical vestibular evoked myogenic potentials among PLD users exceeding the DRC, indicating saccular involvement. There are a few animal studies supporting this finding: Ylikoski [30] reported minimal saccular and utricular macula harm but severe ampullary cristae damage in guinea pigs exposed to intense firearm impulse noise [2]. Akdoğan et al. [31] noted deteriorated epithelial cells, layer separation, and significant crystolysis in guinea pigs saccular maculae after prolonged (6 h) continuous high-intensity noise (120 dB SPL) but not shorter durations. Conversely, Fetoni et al. [32] demonstrated that while intense noise (120 dB SPL for 60 min) caused severe hearing loss, it resulted in only minor vestibular damage, transient VOR gain decreases, and the overexpression of oxidative stress markers. These discrepancies in findings likely stem from variations in SPLs and exposure times across studies.

Current evidence regarding the sound sensitivity of vestibular end-organs is conflicting; some suggest that all five are stimulated by sound stimuli, whereas others point solely to the saccule's sensitivity [32–35]. Semicircular canals are generally considered less susceptible to impulse noise than saccules [33, 36]. However, Maxwell et al. [37] reported high sound pressure levels within semicircular canals during sound stimulation, suggesting similar acoustic energy transmission to both canals and the cochlea. Given that intra-labyrinthine pressures are greater than intracochlear pressures are, high-intensity acoustic stimuli that damage the auditory system might also harm vestibular end-organs [10]. Conversely, low-intensity sounds below the damage risk threshold are less likely to cause semicircular canal injury. In our study, the experimental group's mean PLD output dBA near the tympanic membrane was 69.60 (± 7.88), suggesting a low likelihood of semicircular canal damage. This aligns with the lack of discernible changes in vHIT findings observed in both the control and experimental groups.

Stewart et al. [38] demonstrated differential responses of cochlear and vestibular end-organs in rats to long-term broad-band noise. While hearing loss (approximately 40 dB increase in ABR) was induced, the vestibular system showed 30–60% stereocilia bundle loss in the saccule, utricle, and anterior and horizontal semicircular canals but not in the posterior canal. This correlated with lower baseline firing rates in otolith and anterior semicircular canal afferents & with gain and phase changes in anterior and horizontal semicircular canal afferent responses to head rotation. Notably, no change in rotational or translational VOR gain or phase was observed. The authors attributed the discrepancy between significant stereocilia loss and minimal afferent firing/head rotation changes to vestibular system redundancy, with VOR preservation potentially resulting from the convergence of VOR pathways, an intact contralateral labyrinth, and/or central compensation.

The catchup saccades can be analysed for synchronous sequential refixation by measuring saccade gathering (low PR scores) or scattering (high PR scores). These dispersed and collected responses are correlated with low (uncompensated) and good (well-compensated) compensation, respectively [39]. Because the vestibular and saccadic systems are closely related, saccade incidence, amplitude, latency, and the PR score tend to decrease as VOR gain increases. A lower PR score was associated with better central vestibular compensation. The PR score can be a standalone measure independent of VOR gain to reflect central vestibular compensation [40]. In the case of normal VOR gain, the covert saccades are preserved to stabilize the gaze on the image; hence, normal PR scores are observed. The current study revealed very low PR scores with a low number of covert saccades. Patients with severe vestibular pathology with low VOR gain and no saccades usually have higher PR scores. Notably, the majority of our participants (both experimental and control) had a “clinically normal” VOR gain; hence, lower PR scores and covert saccades were observed in both groups. The statistical analysis revealed no statistically significant difference between the PR scores and cover saccades in either the experimental or the control group. This may be attributed to the lower dBA values used to listen to PLDs. These lower levels may not stimulate the semicircular canals and hence no abnormality in the vHIT findings of both groups.

The current study aimed to position the possible damage to the SCC due to PLD usage. However, no damage to the SCC was observed due to the low volume levels used to listen to PLDs. Nevertheless, recreational noise is a type of noise can also cause a similar alteration to the inner ear, similar to any other type of noise exposure. Thus, damage to the SCC may occur if the levels used to listen to PLDs are high or if the exposure is continued for a longer duration. However, the study recruited relatively smaller sample size for drawing generalizable conclusions. Additionally, potential compensatory mechanisms of the central nervous system were not evaluated separately.

## Conclusion

In conclusion, the present study found no indication of semicircular canal dysfunction among long-term PLD users, despite their significantly higher sound exposure levels, as the measured output levels remained under established DRC. Both groups' VOR gain values and PR scores parameters remain within normal ranges, indicating that low-level PLD use has no negative effects on SCC function.

This study opens avenues for further research into vestibular dysfunction in PLD users. Future studies should include larger samples and examine the long-term cumulative effects of noise, particularly in users exceeding DRC levels or with additional risk factors.

## Data Availability

The data is available in a google spreadsheet on request.
